# Design, Synthesis and Evaluation of Substituted Aryl-2-Nitrovinyl Derivatives as Small Molecules Proteasome Inhibitors

**Published:** 2018

**Authors:** Masoud Faghih Akhlaghi, Marjan Daeihamed, Seyed Abdolmajid Ayatollahi, Farzad Kobarfard, Athar Ata

**Affiliations:** a *Department of Medicinal Chemistry, School of Pharmacy, Shahid Beheshti University of Medical Sciences, Iran. *; b *Department of Pharmaceutics, School of Pharmacy, Guilan University of Medical Sciences, Rasht, Iran. *; c *Phytochemistry Research Center, Shahid Beheshti University of Medical Sciences, Tehran, Iran.*; d * Department of Chemistry, Richardson College of the Environmental Science Complex, The University of Winnipeg, Winnipeg, Canada.*

**Keywords:** Proteasome inhibitor, Small molecule, α, β-unsaturated nitro, Michael acceptor, Aryl-2-nitrovinyl

## Abstract

Based on the existing structure activity relationship for proteasome inhibitors, a number of substituted aryl-2-nitrovinyl derivatives have been synthesized as Michael acceptor and their cytotoxicity and proteasome inhibitory effects were evaluated on two cancer cell lines. Compound 2d exhibited IC_50_ values of 0.71 and 17.79 μM comparable to bortezomib against MCF-7 and PC-3, respectively. The results show that the electronic properties and steric hindrance can affect the interaction of these small molecules with their receptor at the active site of the enzyme while the presence of CH_2_OH group on α-carbon of Michael acceptor is favorable, and para substitution of OMe on phenyl ring of β-carbon can increase the inhibitory potencies. Molecular docking studies confirm our experimental findings about mode of binding of our compounds with 20S proteasome.

## Introduction

The ubiquitin-proteasome system (UPS) is the major intracellular protein degradation pathway in eukaryotic cells. This regulated pathway is responsible for more than 80% of intracellular protein degradation and controls most of the key cellular functions (1, 2). Studies have shown the essential role of UPS in most vital cellular functions, such as cell cycling, signal transduction, immune responses, and apoptosis (3). Protein degradation in the UPS consists of two major steps: the modification of a substrate by addition of poly ubiquitin chain; and subsequent degradation of ubiquitinated protein by 26S proteasome which is a multicatalytic proteinase complex (4, 5). Because of the functional role of this enzyme in cell survival, it has emerged as a promising therapeutic target and proteasome inhibitors have been investigated extensively as potential antitumor, antiviral, anti-inflammatory, and immunosuppressive agents. Furthermore, studies have indicated that tumor cells are more sensitive to proteasome inhibitors than normal cells, and therefore, the proteasome is regarded as one of the most promising antitumor drug targets (6, 7).

The 26S proteasome is a large multi catalytic protein complex, consisting of a cylindrical 20S proteolytic core and two 19S regulatory particles. The 20S core which is responsible for protein degradation comprises of 2 pairs of 14 different subunits and the active sites of the 20S proteasome are located on the β1, β2, and β5 subunits, with caspase-like (C-L), trypsin-like (T-L), and chymotrypsin-like (ChT-L) activities, respectively (3). In all three β-subunits the cleavage of peptide bonds occurs through a mechanism in which the hydroxyl group of N-terminal catalytic threonine residue (Thr1) serves as the nucleophile (2). 

The high rate of protein production in cancer cells and the need for proteasome function in these cells for keeping up the cancer cell survival and proliferation, makes the proteasome pathway a validated target for cancer therapy. After the FDA approval of bortezomib, as a clinically effective reversible inhibitor of the β5 subunit, various proteasome inhibitors have been studied to combat the existing limitations. However, investigations for safer and less toxic alternatives with better pharmacokinetic properties are proceeding (8). Most proteasome inhibitors are composed of a short peptide backbone with an electrophilic trap to capture the hydroxyl group of the N-terminal threonine active site (9). However, these peptide-based drugs are susceptible to degradation and pose short *in-vivo *half-life. As peptide-based drugs suffer from some other limitations like chemical instability, immunogenicity and poor membrane permeability, efforts have been made to develop non-peptide synthetic proteasome inhibitors in the recent years (10). 

The majority of proteasome inhibitors bear an electrophilic trap like epoxyketone, boronic acid, and aldehyde functional groups in their structures (11). In 1997 Bogyo *et al.* (12) introduced a new class of proteasome inhibitors with vinyl sulfone structure acting as a Michael acceptor pharmacophore. Furthermore, Overkleeft *et al.* (13) proposed various Michael acceptor containing peptide-based structures as proteasome inhibitors. On the other hand, studies of Baldisserotto *et al.* (14) showed that compounds bearing α,β-dehydro-phenylalanine are good substrates for Michael addition for catalytic threonine. In sight of these facts and aiming to find non-peptide small molecules, we have considered the synthesis and evaluation of proteasome inhibitory activity of small molecules bearing an α, β-unsaturated nitro functional group as a Michael acceptor in their structure. In this study, a series of substituted aryl-2-nitrovinyl derivatives were designed and synthesized. Docking studies were conducted for the synthesized compounds and the biological activities were also evaluated and compared to a known proteasome inhibitor, bortezomib.

## Experimental

*General methods*

All the chemicals and solvents were purchased from Merck (Darmstadt, Germany). The melting points of the compounds were measured on 9100 Electrothermal melting point apparatus. Elemental analyses were performed by a Costech ECS 4010 CHNS analyzer. The ^1^H-NMR spectra were recorded on a 400 MHz Bruker spectrometer. IR spectra were recorded on Perkin Elmer IR spectrophotometer as potassium bromide discs. GC–MS analysis were carried out by using a 7000 Agilent triple Quadrupole MS system coupled with a 7890A GC, equipped with a split/splitless injection port, an autosampler model Agilent 7693, and electronic ionization. A HP-5MS 5% Phenyl Methyl Silox, Agilent 19091s-433 capillary column was used (30 m × 0.25 mm I.D. and 0.25 μm film thickness). Helium gas with a purity of 99.99% and a flow rate of 1 mL/min was used as the carrier gas.

*General procedure for the synthesis of para-substituted nitrostyrene (1a-e)*

Nitromethane (0.1 moles) and the appropriate aldehyde (0.1 moles) were dissolved in 20 mL of methanol in a beaker that was kept cool by a mixture of ice and salt. A cold solution of sodium hydroxide (10 mL, 0.2 M) was added from an addition funnel to the stirring solution of nitromethane and benzaldehyde at a rate that the temperature was kept below 15 °C. A precipitate was formed and got thick during the addition of sodium hydroxide solution. After fifteen minutes remaining in the lab, about 60 mL of ice water was added to the mixture and the pasty mass was converted to a clear solution. The reaction mixture was added dropwise to a solution of HCl (20 mL of concentrated HCl diluted to 50 mL with water). A solid crystalline mass was formed and separated by decantation. The residue was filtered and washed with water and recrystallized from ethanol (15). 

(2-nitrovinyl)benzene (1a): Yellow solid. Yield: 83%. mp 57-58 °C. IR (KBr): 1635, 1523, 1348, 973, 778. GC-MS (m/z): 149.1 [M]^+^.^ 1^H-NMR (400 MHz, CDCl_3_): 7.43-7.56 (5H, m, CH_Ph_), 7.59 (1H; d, J = 13.7 Hz; =CH_α_), 8.01 (1H; d, J = 13.7 Hz; =CH_β_). Anal. Calcd. For C_8_H_7_NO_2_ (149.15): C, 64.42; H, 4.73; N, 9.39; O, 21.45. Found: C, 64.37; H, 4.69; N, 9.41; O, 21.38.

1-chloro-4-(2-nitrovinyl)benzene (1b): Crystalline (needle shape), light yellowish solid. Yield: 46%. mp 111-113 °C. IR (KBr): 1627, 1587, 1525, 1343, 979. GC-MS (*m/z*): 183.0 [M]^+^. ^1^H-NMR (400 MHz, CDCl_3_): 7.44 (2H; d, J = 8.6 Hz; CH_Ar-O-Cl_)_, _7.49 (2H; d, J = 8.6 Hz; CH_Ar-m-Cl_), 7.56 (1H; d, J = 13.7 Hz; =CH_α_), 7.96 (1H; d, J = 13.7 Hz; =CH_β_). Anal. Calcd. For C_8_H_6_ClNO_2_ (183.59): C, 52.34; H, 3.29; Cl, 19.31; N, 7.63; O, 17.43. Found: C, 52.32; H, 3.30; Cl, 19.30; N, 7.63; O, 17.41.

1-methyl-4-(2-nitrovinyl)benzene (1c): Yellow solid. Yield: 57%. mp 105-106 °C; IR (KBr): 1628, 1514, 1345, 967, 815. GC-MS (m/z): 163.1 [M]^+^. ^1^H-NMR (400 MHz, CDCl_3_): 2.41 (3H, s, Me), 7.26 (2H; d, J = 8Hz; CH_Ar-o-Me_), 7.45 (2H; d, J = 8Hz; CH_Ar-m-Me_), 7.57 (1H; d, J = 13.6 Hz; =CH_α _), 7.99 (1H; d, J = 13.6 Hz; =CH_β_). Anal. Calcd. For C_9_H_9_NO_2_ (163.17): C, 66.25; H, 5.56; N, 8.58; O, 19.61. Found: C, 66.22; H, 5.57; N, 8.56; O, 19.60.

**Scheme 1:**
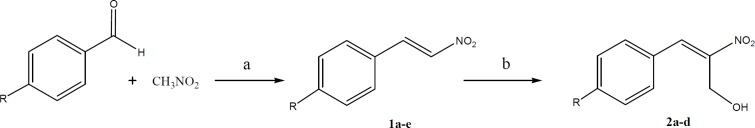
The synthetic pathway for preparation of para-substituted nitrostyrenes (1a-e) and 2-nitro-3-arylprop-2-en-1-ols (2a-d). Reagents and conditions: a) NaOH, temp. < 15 °C; b) Imidazole, anthranilic acid, formaldehyde solution, r. t

**Scheme 2:**
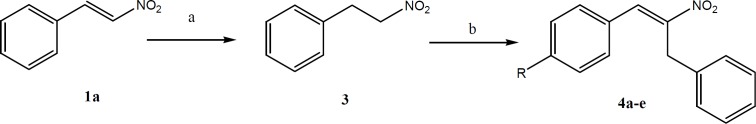
The synthetic pathway for preparation of aryl-2-nitroallylbenzene (4a-e). Reagents and conditions: a) Silica gel, NaBH_4_, 2-propanol and chloroform; b) Dimethylamine hydrochloride, potassium fluoride, toluene.

**Scheme 3:**
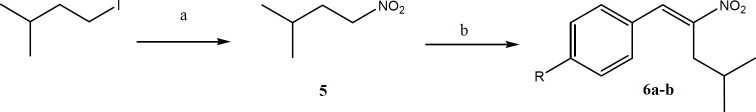
The synthetic pathway for preparation of Aryl-4-methyl-2-nitropent-1-ene (6a-b). Reagents and conditions: a) Isopentyl iodide, sodium nitrite, DMF, 0 °C; b) n-BuNH_2_, methanol and acetic acid, 0 °C, under argon.

**Figure 1:**
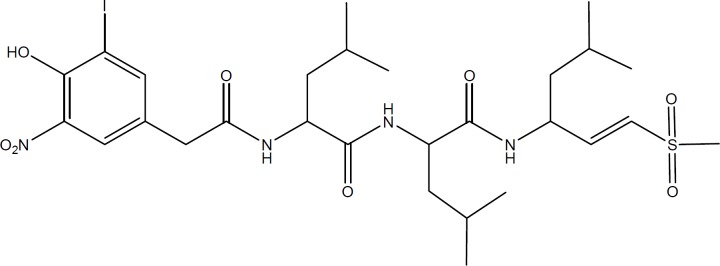
Chemical structure of NIP-Leu_3_-Vinyl sulfone as a representative of vinyl sulfones

**Figure 2:**
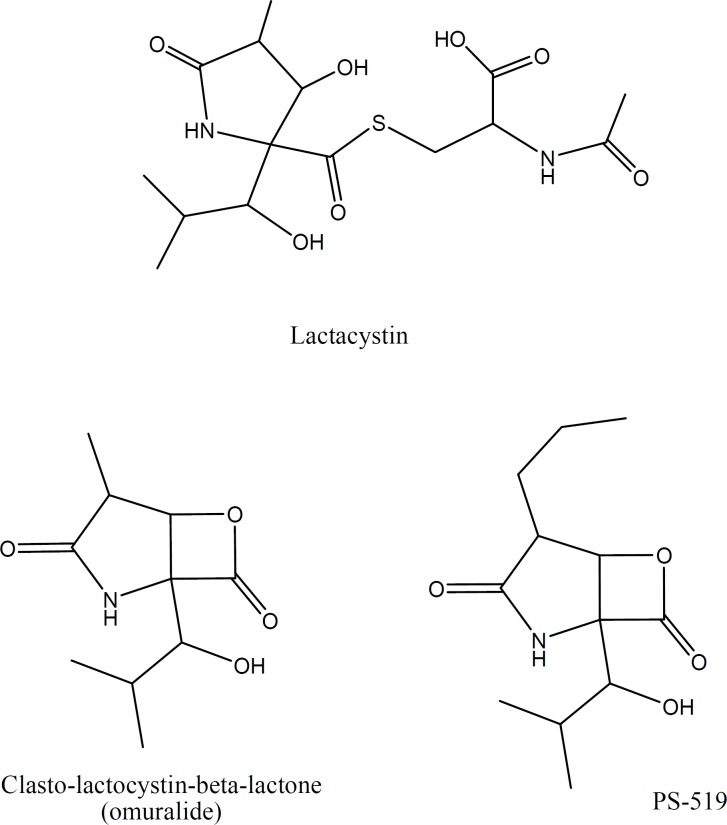
Chemical structure of Lactacystin and its derivatives

**Figure 3:**
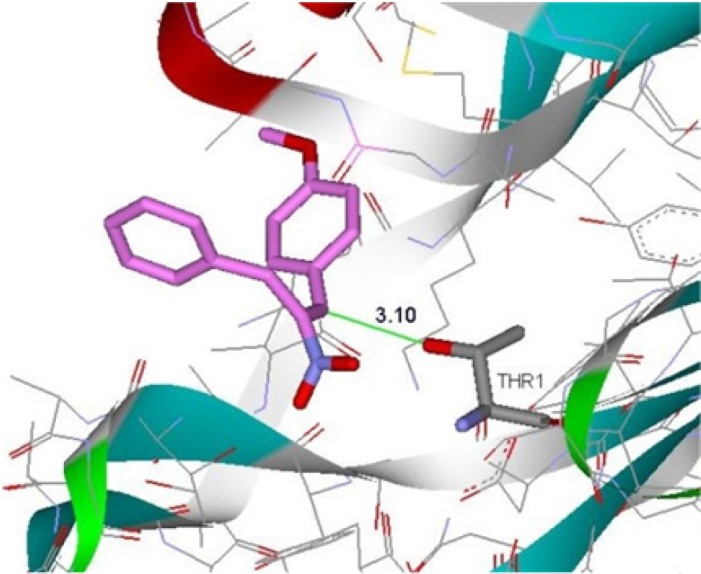
Binding of compound 4d in the active site of β5-subunit of 20S proteasome

**Table 1:** Cytotoxicity and proteasome ChT-L inhibitory activities of substituted aryl-2-nitrovinyl derivatives on MCF-7 and PC-3 cell lines.

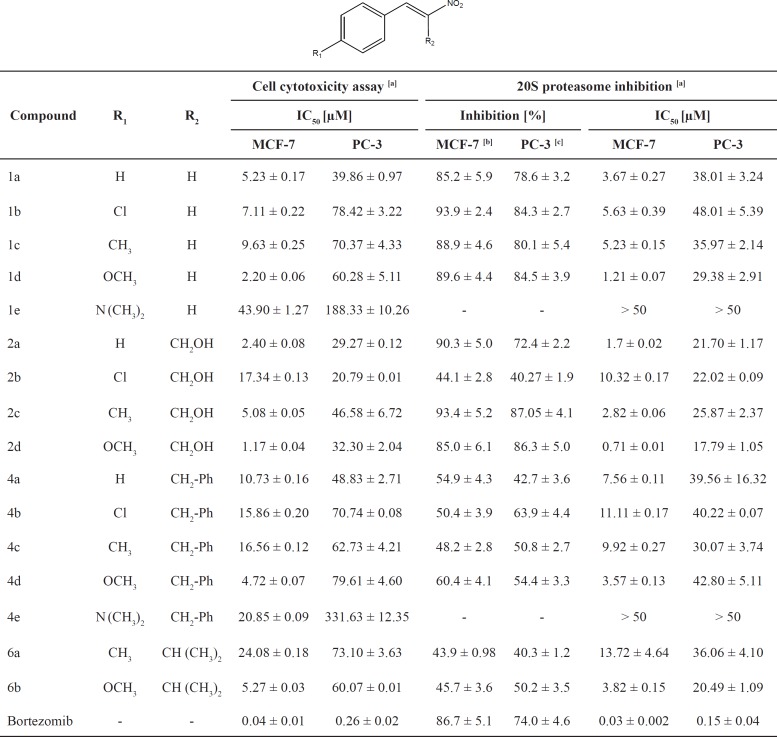

^[a]^Values represent the mean ± SD of three independent experiments, each based on four biological replicates. ^[b]^Percent inhibition at 10 µM. ^[c]^ Percent inhibition at 25 µM.

1-methoxy-4-(2-nitrovinyl) benzene (1d): Crystalline (needle shape) yellow solid. Yield: 96%. mp 84-86 °C; IR (KBr): 1624, 1509, 1419, 1324, 973. GC-MS (m/z): 179.1 [M]^+^. ^1^H-NMR (400 MHz, CDCl_3_): 3.87 (3H, s, OMe), 6.95 (2H; d, J = 8.8 Hz; CH_Ar-o-OMe_), 7.49-7.54 (3H, m, CH_Ar-m-OMe_ and =CH_α_), 7.97 (1H; d, J = 13.6 Hz; =CH_β_). Anal. Calcd. For C_9_H_9_NO_3_ (179.17): C, 60.33; H, 5.06; N, 7.82; O, 26.79. Found: C, 60.31; H, 5.08; N, 7.80; O, 26.79.

N, N-dimethyl-4-(2-nitrovinyl)benzenamine (1e): Red solid. Yield: 30%. mp 182-184 °C; IR (KBr): 1668, 1547, 1493, 1339, 981. GC-MS (m/z): 192.1 [M]^+^. ^1^H-NMR (400 MHz, CDCl_3_): 3.08 (6H, s, CH_N(Me)2_), 6.9 (2H; d, J = 8.9 Hz; CH_Ar-o-N(Me)2_), 7.43 (2H; d, J = 8.9 Hz; CH_Ar-m-N(Me)2_), 7.50 (1H, d, J = 13.4 Hz; =CH_α_), 7.97 (1H, d, J = 13.4 Hz; =CH_β_). Anal. Calcd. For C_10_H_12_N_2_O_2_ (192.21): C, 62.49; H, 6.29; N, 14.57; O, 16.65. Found: C, 62.48; H, 6.29; N, 14.55; O, 16.66.

*General procedure for the synthesis of 2-nitro-3-arylprop-2-en-1-ol (2a-d)*

To a stirred solution of substituted nitrostyrene (10 mmol) in THF (25 mL) at room temperature, imidazole (1 equivalent) and anthranilic acid (10 mol%) were added. Aqueous solution of formaldehyde (37%, 25 mL) was then added and the mixture was stirred at room temperature overnight. After completion of the reaction (monitored by TLC), the mixture was acidified with 5 M HCl (20 mL) and the aqueous layer was extracted with EtOAc (3 × 25 mL). The organic layers were washed with brine (50 mL), dried with anhydrous Na_2_SO_4_, and concentrated .The residue was purified by column chromatography (silica gel, EtOAc–hexanes, 0–30%, gradient elution) to obtain pure product (16).

2-Nitro-3-phenylprop-2-en-1-ol (2a): Yellow oil. Yield: 53%. IR (neat): 3437, 1658, 1531, 1238, 1027, 783. GC-MS (m/z): 179.1 [M]^+^. ^1^H-NMR (400 MHz, CDCl_3_): 2.67 (1H, s, br), 4.72 (2H, s, CH_2_), 7.48-7.58 (5H, m, CH_Ph_), 8.22 (1H, s, =CH). Anal. Calcd. For C_9_H_9_NO_3_ (179.17): C, 60.33; H, 5.06; N, 7.82; O, 26.79. Found: C, 60.35; H, 5.06; N, 7.79; O, 26.80.

2-Nitro-3-(4-chlorophenyl)prop-2-en-1-ol (2b): Pale yellow oil. Yield: 32%. IR (neat): 3441, 1636, 1506, 1453, 1091, 841. GC-MS (m/z): 213.0 [M]^+^. ^1^H-NMR (400 MHz, CDCl_3_): 2.66 (1H, s, br), 4.67 (2H, s, CH_2_), 7.46 (2H; d, J = 8 Hz; CH_Ar-o-Cl_), 7.52 (2H; d, J = 8 Hz; CH_Ar-m-Cl_), 8.16 (1H, s, =CH). Anal. Calcd. For C_9_H_8_ClNO_3_ (213.62): C, 50.60; H, 3.77; Cl, 16.60; N, 6.56; O, 22.47. Found: C, 50.59; H, 3.78; Cl, 16.62; N, 6.54; O, 22.46.

2-Nitro-3-(4-tolyl)prop-2-en-1-ol (2c): Yellow solid. Yield: 38%. mp 52–54 °C. IR (KBr): 3444, 1660, 1539, 1342, 1030, 833. GC-MS (m/z): 193.1 [M]^+^. ^1^H-NMR (400 MHz, CDCl_3_): 2.42 (3H, s, Me), 2.67 (1H, s, br), 4.71 (2H; s, CH_2_), 7.29 (2H; d, J = 8 Hz; CH_Ar-o-Me_), 7.47 (2H; d, J = 8 Hz; CH_Ar-m-Me_), 8.19 (1H, s, =CH). Anal. Calcd. For C_10_H_11_NO_3 _(193.2): C, 62.17; H, 5.74; N, 7.25; O, 24.84. Found: C, 62.15; H, 5.74; N, 7.23; O, 24.85.

2-Nitro-3-(4-methoxyphenyl)prop-2-en-1-ol (2d): Yellow solid. Yield: 54%. mp 71–73 °C. IR (KBr): 3424, 1636, 1512, 1382, 1024, 831. GC-MS (m/z): 209.1 [M]^+^. ^1^H-NMR (400 MHz, CDCl_3_): 2.62 (1H, s, br), 3.48 (3H, s, OMe), 4.73 (2H, s, CH_2_), 6.99 (2H; d, J = 8 Hz; CH_Ar-o-OMe_), 7.50 (2H; d, J = 8 Hz; CH_Ar-m-OMe_), 8.19 (1H, s, =CH). Anal. Calcd. For C_10_H_11_NO_4_ (209.2): C, 57.41; H, 5.30; N, 6.70; O, 30.59. Found: C, 57.43; H, 5.31; N, 6.69; O, 30.60.

*Synthesis of (2-nitroethyl)benzene (3)*

To the stirred mixture of nitrostyrene (1a, 10 mmol), silica gel (20 g, column chromatography grade), 2-propanol (30 mL), and chloroform (160 mL) was added NaBH_4_ (40 mmol) in small portions over a period of 15 min at room temperature. The mixture was stirred for additional 15 min until the yellow color was completely disappeared. Excess NaBH_4_ was decomposed by addition of diluted HCl (0.1 N) and the mixture was filtered and washed with CH_2_C1_2_. The combined filtrate was washed with brine, dried (Na_2_SO_4_) and then evaporated to give (2-nitroethyl)benzene as a clear oil (17).

IR (neat): 3063, 3031, 1608, 1555, 1380, 752. ^1^H-NMR (400 MHz, CDCl_3_): 5.44 (2H, s, CH_2_), 7.45-7.50 (5H, m, CH_Ph_). Anal. Calcd. For C_8_H_9_NO_2 _(151.16): C, 63.56; H, 6.00; N, 9.27; O, 21.17. Found: C, 63.55; H, 6.02; N, 9.26; O, 21.14.

*General procedure for the synthesis of aryl-2-nitroallylbenzene (4a-4e)*

A mixture of (2-nitroethyl)benzene (15 mmol), dimethylamine hydrochloride (30 mmol), potassium fluoride (2.25 mmol) and appropriate aldehyde (15 mmol) in toluene (20 mL) was refluxed with a Dean–Stark trap for 24 h. The reaction mixture was then diluted with toluene (100 mL) and washed with 10% HCl solution (3 × 30 mL). The organic phase was dried over anhydrous Na_2_SO_4_ and solvent evaporated *in-vacuo*. The product was purified by recrystallization in a mixture of EtOAc/hexane or column chromatography over silica gel (EtOAc/hexane, 7/3) (18).

3-(phenyl)-2-nitroallylbenzene (4a): Yellow oil. Yield: 67%. IR (neat): 1649, 1533, 1392, 1331, 779. GC-MS (m/z): 239.1 [M]^+^. ^1^H-NMR (400 MHz, CDCl_3_): 4.27 (2H, s, CH_2_), 7.21-7.27 (5H, m, CH_benzyl ring_), 7.42-7.47 (5H, m, CH_Ph_), 8.31 (1H, s, =CH). Anal. Calcd. For C_15_H_13_NO_2_ (239.27): C, 75.30; H, 5.48; N, 5.85; O, 13.37. Found: C, 75.28; H, 5.49; N, 5.83; O, 13.35.

3-(4-chlorophenyl)-2-nitroallylbenzene (4b): Light brown solid. Yield: 78%. mp 98–100 °C. IR (KBr): 1651, 1520, 1325, 1021, 877. GC-MS (m/z): 273.1 [M]^+^. ^1^H-NMR (400 MHz, CDCl_3_): 4.22 (2H, s, CH_2_), 7.14 (2H; d, J = 8.4 Hz; CH_Ar-o-Cl_), 7.29 (2H; d, J = 8.4 Hz; CH_Ar-m-Cl_), 7.42-7.44 (5H, m, CH_Ph_), 8.31 (1H, s, =CH). Anal. Calcd. For C_15_H_12_ClNO_2_ (273.71): C, 65.82; H, 4.42; Cl, 12.95; N, 5.12; O, 11.69. Found: C, 65.80; H, 4.40; Cl, 12.94; N, 5.14; O, 11.70.

3-(4-methylphenyl)-2-nitroallylbenzene (4c): Yellow-green solid. Yield: 73%. mp 105–107 °C. IR (KBr): 1665, 1520, 1393, 1329, 782. GC-MS (m/z): 253.1 [M]^+^. ^1^H-NMR (400 MHz, CDCl_3_): 2.32 (3H, s, Me), 4.22 (2H, s, CH_2_), 7.09-7.14 (4H, m, CH_Ar-o-Me_ and CH_Ar-m-Me_), 7.40-7.46 (5H, m, CH_Ph_), 8.28 (1H, s, =CH). Anal. Calcd. For C_16_H_15_NO_2_ (253.3): C, 75.87; H, 5.97; N, 5.53; O, 12.63. Found: C, 75.88; H, 5.96; N, 5.53; O, 12.61.

3-(4-methoxyphenyl)-2-nitroallylbenzene (4d): Yellow solid. Yield: 57%. mp 53–56 °C. IR (KBr): 1661, 1523, 1342, 1303, 878. GC-MS (m/z): 269.1 [M]^+^. ^1^H-NMR (400 MHz, CDCl_3_): 3.85 (3H, s, OMe), 4.31 (2H, s, CH_2_), 6.95 (2H; d, J = 8 Hz; CH_Ar-o-OMe_), 7.20-7.39 (5H, m, CH_Ph_), 7.45 (2H; d, J = 8 Hz, CH_Ar-m-OMe_), 8.35 (1H, s, =CH). Anal. Calcd. For C_16_H_15_NO_3_ (269.3): C, 71.36; H, 5.61; N, 5.20; O, 17.82. Found: C, 71.35; H, 5.62; N, 5.23; O, 17.80.

N, N-dimethyl-4-(2-nitro-3-phenylprop-1-enyl)benzenamine (4e): Dark red solid. Yield: 76%. mp 110–113 °C. IR (KBr): 1599, 1530, 1386, 1252, 875. GC-MS (m/z): 282.1 [M]^+^. ^1^H-NMR (400 MHz, CDCl_3_): 3.02 (6H, s, N(Me)_2_), 4.34 (2H, s, CH_2_), 6.65 (2H; d, J = 9 Hz; CH_Ar-o-N(Me)2_), 7.22-7.34 (5H, m, CH_Ph_), 7.40 (2H; d, J = 9 Hz, CH_Ar-m- N(Me)2_), 8.35 (1H, s, =CH). Anal. Calcd. For C_17_H_18_N_2_O_2_ (282.34): C, 72.32; H, 6.43; N, 9.92; O, 11.33. Found: C, 72.30; H, 6.44; N, 9.94; O, 11.32.

*Synthesis of 3-methyl-1-nitrobutane (5)*

Isopentyl iodide (50.0 mmol) dissolved in DMF (40 mL) was added dropwise to a stirred solution of DMF (150 mL) and sodium nitrite (100.0 mmol) at 0 °C. The mixture was stirred overnight at room temperature. Water (500 mL) was then added to the mixture and it was extracted with petroleum ether (4 × 70 mL). The combined organic phase was washed with water (2 × 50 mL) and dried with Na_2_SO_4_. The solvent was evaporated under reduced pressure to give 3-methyl-1-nitrobutane as colorless oil. 

IR (neat): 2962, 2870, 1785, 1555, 1473, 1275, 1135. ^1^H-NMR (400 MHz, CDCl_3_): 0.89 (6H; d, J = 6.6 Hz; -CH_3-isopropyl_), 1.58-1.66 (1H, m, -CH-_isopropyl_), 1.85 (2H; q, J = 7.3 Hz; -CH_β_), 4.34 (2H; t, J = 7.3 Hz, CH_2_). Anal. Calcd. For C_5_H_11_NO_2_ (117.15): C, 51.26; H, 9.46; N, 11.96; O, 27.32. Found: C, 51.27; H, 9.46; N, 11.95; O, 27.30.

*General procedure for the synthesis of Aryl-4-methyl-2-nitropent-1-ene (6a-b)*

To a stirred solution of 3-methyl-1-nitrobutane (28 mmol) and appropriate aldehyde (28 mmol) in MeOH (20 mL), AcOH (1.08 mL, 18.8 mmol) and n-BuNH_2_ (1.86 mL, 18.8 mmol) were added at 0 °C under argon. After being stirred for 36 h at room temperature, the reaction mixture was poured into water and then extracted with EtOAc. The organic layer was washed with water and brine, dried over Na_2_SO_4_ and concentrated. The resultant residue was purified by column chromatography on silica gel (EtOAc/hexane, 1/20) (19).

1-methyl-4-(4-methyl-2-nitropent-1-enyl)benzene (6a): Colorless oil. Yield: 40%. IR (neat): 1640, 1524, 1384, 1188, 770. GC-MS (m/z): 219.1 [M]^+^. ^1^H-NMR (400 MHz, CDCl_3_): 0.96 (6H; d, J = 6.8 Hz; -(CH_3_)_2-isopropyl_), 2.1 (1H; n, J = 6.8 Hz; -CH-_isopropyl_), 2.40 (3H, s, Me), 2.83 (2H; d, J = 7.2 Hz; CH_2_), 7.25 (2H; d, J = 8 Hz; CH_Ar-o-Me_), 7.36 (2H; d, J = 8 Hz; CH_Ar-m-Me_), 8.04 (1H, s, =CH). Anal. Calcd. For C_13_H_17_NO_2_ (219.28): C, 71.21; H, 7.81; N, 6.39; O, 14.59. Found: C, 71.19; H, 7.80; N, 6.39; O, 14.60.

1-methoxy-4-(4-methyl-2-nitropent-1-enyl)benzene (6b): Yellow oil. Yield: 44%. IR (neat): 1599, 1506, 1302, 1025, 832. GC-MS (m/z): 235.1 [M]^+^. ^1^H-NMR (400 MHz, CDCl_3_): 0.95 (6H; d, J = 6.8 Hz; -(CH_3_)_2-isopropyl_), 2.0 (1H; n, J = 6.8 Hz; -CH-_isopropyl_), 2.42 (2H; d, J = 7.2 Hz; CH_2_), 3.86 (3H, s, OMe), 6.97 (2H; d, J = 8 Hz; CH_Ar-o-OMe_), 7.42 (2H; d, J = 8 Hz; CH_Ar-m-OMe_), 8.05 (1H, s, =CH). Anal. Calcd. For C_13_H_17_NO_3 _(235.28): C, 66.36; H, 7.28; N, 5.95; O, 20.40. Found: C, 66.35; H, 7.29; N, 5.96; O, 20.39.

*Cell viability assays*

Human breast cancer (MCF-7), and human prostate cancer (PC-3) cells were cultured in their respective media supplemented with 10% fetal bovine serum (FBS). The cells were cultured at 1 × 10^4^ cells per well, in a 96-well plate and incubated for 24-48 h to obtain cell attachments. Afterwards, the cells were treated with different concentrations of test compounds for 48 h and then incubated with 20 µL of 5 mg/mL 3-(4,5-dimethylthiazol-2-yl)-2,5-diphenyl tetrazolium bromide (MTT) solution for 4 h. After aspiration of the MTT solution, 100 µL of DMSO was added to each well to dissolve formazan crystals. The absorbance of each well was measured at the wavelength of 550 nm using an enzyme-linked immunosorbent assay reader. IC_50 _values were calculated from the curves generated by plotting the percentage of the viable cells versus the test concentration on a logarithmic scale using SigmaPlot 10.0 software.

*Cell-based proteasome inhibition assay*

The effect of the derivatives on the 20S proteasome activity was determined using the 20S proteasome activity assay kit from Sigma (USA). This assay measures the ChT-L protease activity in cultured cells and is based on the detection of the fluorophore R110 after cleavage from the labeled substrate LLVY-R110. MCF-7 and PC-3 cells were pretreated with different concentrations of corresponding compounds and proteasome activities were determined according to the manufacturer′s protocol. Proteasome activity generates strongly green fluorescent R110, which was monitored at an excitation wavelength of 480 nm and emission of 590 nm on a microplate fluorescence reader. All data were calculated by subtracting the blank control. The data are presented as percent inhibition at a specific concentration (10 µM for MCF-7 and 25 µM for PC-3), and IC_50_ values obtained from dose–response curves utilizing curve-fitting software package SigmaPlot 10.0 software.

*Docking studies*

The crystal structure of 20S proteasome (PDB code: 4INR) was obtained from the RCSB Protein Data Bank. The molecular docking study was performed using AutoDock Vina v.1.1.2. Protein was prepared for docking by removing co-crystallized ligand and all water molecules from crystal protein. Polar hydrogens were added and nonpolar hydrogens were merged, finally Kallman unitedatom charge and atom type parameter were added to 4INR. Grid box dimensions (20 × 20 × 20) were set surrounding active site (20). 

## Results and Discussion

*Chemistry*

The routes of synthesis for substituted aryl-2-nitrovinyl derivatives are outlined in Schemes 1–3. The para-substituted nitrostyrenes (1a-e) were prepared by the reaction of nitromethane with the appropriate aldehyde. The obtained substituted nitrostyrenes (1a-d) were then converted to 2-nitro-3-arylprop-2-en-1-ols (2a-d) in the presence of imidazole, anthranilic acid, and formaldehyde solution at room temperature according to the synthetic route shown in Scheme 1.

For synthesis of aryl-2-nitroallylbenzenes (4a-e), first (2-nitroethyl) benzene (3) was prepared by reduction of nitrostyrene (1a) in the presence of silica gel and NaBH_4_ in 2-propanol and chloroform. After that, compound 3 was reacted with appropriate aldehyde by adding dimethylamine hydrochloride and potassium fluoride in toluene.

Aryl-4-methyl-2-nitropent-1-ene (6a-b) was synthesized by the reaction of 3-methyl-1-nitrobutane (5) with the appropriate aldehyde in methanol, in the presence of n-BuNH_2_ and acetic acid at 0 °C and under argon. 3-methyl-1-nitrobutane (5) was prepared by the reaction of isopentyl iodide with sodium nitrite in DMF at 0 °C.

*Biological evaluation*

Proteasome inhibition have been proposed as a promising therapeutic target in medicinal chemistry and various reversible and irreversible proteasome inhibitors have been introduced in the literature (21). Most proteasome inhibitors are small peptides containing an electrophilic functional group at one end of their structures which form covalent bonds with the catalytic O^γ^-threonine in the active site of 20S proteasome (10). Vinyl sulfones (Figure 1) were introduced as a pharmacophore in irreversible proteasome inhibitors by Bogyo *et al.* (12), and it is suggested that the hydroxyl group of the threonine residue in the active site of proteasome reacts with the double bond of vinyl sulfone moiety through a Michael addition mechanism.

Conjugation of vinyl group with sulfone moiety makes these compounds good Michael acceptors and thus good proteasome inhibitors. In the present study we designed and synthesized a group of nitrovinyl compounds (α, β–unsaturated nitro compounds) which are chemically eligible Michael acceptors and they can then be considered as potential proteasome inhibitors. The reported structure activity relationship for different classes of proteasome inhibitors such as peptide-aldehydes, vinyl sulfones, peptide boronates (bortezomib) and epoxyketones indicate that the presence of an amino acid residue, such as leucine or phenylalanine, in the vicinity of the pharmacophore group will increase the efficiency of proteasome inhibitors. Therefore, we tried three residues next to the nitrovinyl moiety and investigated their impact on proteasome inhibitory activity. The three residues were isopropyl, benzyl and hydroxymethyl, and they were placed on the alpha carbon with respect to the nitro group to avoid any possible steric hindrance on beta carbon.

The synthesized derivatives were tested for the antiproliferative and ChT-L proteasome inhibitory properties on two cancer cell lines including MCF-7 and PC-3 cells. A list of the designed compounds and their biological activity are summarized in Table 1. In order to investigate the nature of the interaction of these small molecules with the receptor, various functional groups with different lipophilicity, size and electronic properties were substituted at the R_1_ (Cl, CH_3_, OCH_3_, and N (CH_3_)_2_) and R_2_ (isopropyl, benzyl, or CH_2_OH) positions.

As shown in Table 1, most of the substituted aryl-2-nitrovinyl derivatives, with the exception of compounds 1e and 4e showed promising inhibitory activities toward ChT-L in both cell lines, inhibiting >40% of the proteasome activity at 10 and 25 µM concentrations for MCF-7 and PC-3, respectively. The 2-nitro-3-(4-methoxyphenyl)prop-2-en-1-ol (compound 2d) was the most potent proteasome inhibitor with IC_50_ values of 0.71 and 17.79 μM against MCF-7 and PC-3, respectively. These α,β–unsaturated nitro compounds also exhibited satisfactory cytotoxic effects against both MCF-7 (IC_50 _< 25) and PC-3 (IC_50 _< 80) cancer cell lines.

Among the R_2_ substituted derivatives, compounds with a CH_2_OH group on the α-carbon position of the nitro group (2a, 2c, 2d), generally showed the highest ChT-L inhibitory effects. These compounds exhibited lower antiproliferative and proteasome inhibitory IC_50_ values on both cell lines. Introducing benzyl ring and isopropyl group at the R_2_ position resulted in lower activities (*i.e.* 4c and 6a versus 2c) and the following order was seen for proteasome inhibitory potencies in the R_2_ position: CH_2_OH > H > CH_2_-Ph > CH (CH_3_)_2_; suggesting that parameters such as steric and electronic properties of the side chain on α-carbon position of the α,β-unsaturated nitro group may affect the activity of the compounds. Despite the fact that the best amino acid residue next to the pharmacophore group in boronates and vinyl sulfones seem to be isobutyl group, the presence of a hydroxymethyl group in the vicinity of pharmacophore moiety is also observed in the structures of Lactacystin (Figure 2) and its derivatives which are natural products with proteasome inhibitory activity.

Maintaining the R_2_ position constant, various para-substituted (2-nitrovinyl)benzene derivatives were synthesized and evaluated for ChT-L inhibitory properties. The presence of OMe group at R_1 _position (1d, 2d, 4d, and 6b) significantly improved the antiproliferative and ChT-L proteasome inhibitory activities in each series. The replacement of OMe with other substituents including Me, Cl, and N (CH_3_)_2 _reduced proteasome inhibitory effects whereas no inhibitory effects (IC_50_ > 50 μM) were observed for 1e and 4e in both cell lines, bearing N (CH_3_)_2_ group at R_1_ position. These results propose that the electronic properties of the substituent and steric hindrance at R_1_ position may have marked effects on ChT-L proteasome inhibitory properties.

*Docking studies*

Molecular docking studies were conducted to understand the interactions between the efficient derivatives and the active site of β5-subunit of the proteasome (Thr1). According to the results of docking studies (Figure 3), the distance between β-position of Michael acceptor and OH group of Thr1 in the active site of enzyme is about 3.1 Ǻ that can suggest the possibility of covalent interactions. The Gibbs free energy binding (ΔG), obtained from the result of molecular docking, showed that the more active derivatives have a satisfactory affinity to β5-subunit of proteasome with free binding energy of –6 kcal/mol.

## Conclusion

In the present study a series substituted aryl-2-nitrovinyl derivatives have been synthesized and their inhibitory activities were assessed against β5-subunit of the 20S proteasome in two cell lines. The tested derivatives inhibited proteasome activity and cell proliferation with good IC_50_ values on both cell lines. Among the tested compounds, compound 2d proved to be the most potent proteasome inhibitor with IC_50_ value of 0.71 and 17.79 μM against MCF-7 and PC-3, respectively.
